# Topological Design of Cellular Phononic Band Gap Crystals

**DOI:** 10.3390/ma9030186

**Published:** 2016-03-10

**Authors:** Yang Fan Li, Xiaodong Huang, Shiwei Zhou

**Affiliations:** 1 Centre for Innovative Structures and Materials, School of Engineering, RMIT University, GPO Box 2476, Melbourne 3001, Australia; s3495356@student.rmit.edu.au (Y.F.L.); shiwei.zhou@rmit.edu.au (S.Z.); 2 Key Laboratory of Advanced Technology for Vehicle Body Design & Manufacture, Hunan University, Changsha 410082, China

**Keywords:** cellular phononic crystals, band gap, homogenization, BESO, bulk modulus, shear modulus

## Abstract

This paper systematically investigated the topological design of cellular phononic crystals with a maximized gap size between two adjacent bands. Considering that the obtained structures may sustain a certain amount of static loadings, it is desirable to ensure the optimized designs to have a relatively high stiffness. To tackle this issue, we conducted a multiple objective optimization to maximize band gap size and bulk or shear modulus simultaneously with a prescribed volume fraction of solid material so that the resulting structures can be lightweight, as well. In particular, we first conducted the finite element analysis of the phononic band gap crystals and then adapted a very efficient optimization procedure to resolve this problem based on bi-directional evolutionary structure optimization (BESO) algorithm in conjunction with the homogenization method. A number of optimization results for maximizing band gaps with bulk and shear modulus constraints are presented for out-of-plane and in-plane modes. Numerical results showed that the optimized structures are similar to those obtained for composite case, except that additional slim connections are added in the cellular case to support the propagation of shear wave modes and meanwhile to satisfy the prescribed bulk or shear modulus constraints.

## 1. Introduction

The propagation of mechanical waves in periodic structures has aroused a growing research interest in recent years. The existence of band gaps in phononic crystals can lead to a variety of potential applications in sound insulation, shock isolations, seismic wave-proofing, acoustic wave filtering, waveguides, negative refraction, *etc.* [1]. In phononic crystals (PnCs), the elastic properties are periodic functions of position with a periodicity comparable to the wavelength of the corresponding wave field [2]. The underlying mechanism for the formation of band gaps relies on the different scattering of a mechanical wave at the interface between constituent phases with high contrast in density and/or elastic constants. In the past few years, most studies consider PnCs consisting of two solid components, solid-fluid or fluid-fluid components. Among these different patterns, cellular PnCs with air or vacuum cylindrical holes embedded in a host material are more economical and practical, as well as multi-functional. Compared with composite PnCs, cellular PnCs can be very light and achieve high toughness, while reducing the fabrication cost. In this research we only consider the case of cellular phononic band gap crystals.

Calculation of band structure for acoustic or elastic waves propagating in periodic composite structures was first carried out by Sigalas *et al.* in 1992 [3] by using plane wave expansion method (PWE), and the concept of PnCs was first conceived by Kushwaha in 1993 [4,5]. Although the PWE method is capable to calculate dispersion relation of mechanical waves, it has convergence and accuracy problems in the case of fluid/solid system, especially for the case when the contrast of material parameters between inclusions and the substrate is very high [2]. Later, various techniques have been successfully introduced to calculate band structure of acoustic and elastic waves in fluid/solid or vacuum/solid PnCs, such as the transfer matrix method (TMM) [6], multiple scattering theory (MST) [7], finite-difference time-domain (FDTD) [8,9,10], Rayleigh method (RM) [11], and finite element method (FEM) [12], *etc*.

The application of phononic band gap crystals highly depends on the width of the band gap and the key issue is therefore to engineer the phononic band gap as wide as possible. A topology optimization method provides an effective means for systematic and scientific approach of designing phononic structures with an optimal band gap. Optimization of phononic band gap crystals was first conducted by Sigmund and Jensen [13] based on finite element method (FEM) in combination with the method of moving asymptotes (MMA). In the following, genetic algorithm (GA) and gradient-based topology optimization, in conjunction with FEM or the fast plane wave expansion method (FPWE), are developed to maximize the band gap sizes of phononic band gap crystals [14,15,16,17,18,19,20]. However, most of the optimization work is conducted for the case of solid composite materials, and few studies focus on the case of air/vacuum inclusions embedded in a host medium [16]. Dong *et al.* [17] conducted a multi-objective optimization of 2D porous phononic crystals for maximizing band gap width and minimizing mass of structure simultaneously by using non-dominated sorting-based genetic algorithm II (NSGA-II). To guarantee the self-support of cellular phononic crystals, an artificial geometrical constraint is adapted.

It is apparent that current research in this area is insufficient and further systematic investigation into the design of cellular phononic band gap crystals is necessary, especially for their multi-functionalities. In order to take advantage of light-weight cellular phononic crystals, mass of structure should be minimized or subjected to a certain constraint. In fact, both optimization results for composite phononic crystals and the existing cellular results indicate that maximizing band gap tends to be the isolation of solid material with high density and stiffness; however, transverse/shear wave is not supported in air/vacuum. Thus, it is meaningful to make sure that the optimized phononic band gap structures have continuous distribution of solid material to sustain static loads. In contrast with simply adding a geometrical constraint to the optimized structures, it is more meaningful to consider the additional functionality of phononic crystals, such as bulk or shear modulus, so that the resulting structures could achieve appropriate stiffness for undergoing external forces. To the authors’ best knowledge, no work has been reported yet to optimize the cellular phononic crystals with a stiffness constraint and volume constraint simultaneously.

The purpose of this research is to maximize the gap size between two appointed bands for cellular phononic crystals subject to bulk or shear modulus constraint with a given volume fraction. The rest of the paper is organized as follows: In Section 2 we introduce the essential governing equations and related theories of phononic crystals, along with the homogenization theory to calculate the effective bulk or shear modulus of cellular structures. Then we formulate the optimization problem into the mathematical equations and present the topology optimization algorithm used in this paper. In Section 3, we discuss the numerical implementations of the optimization problem and the associated sensitivity analysis. A number of numerical examples and optimized cellular phononic structures are presented in Section 4 for out-of-plane waves, in-plane waves, and the coupled in-plane and out-of-plane waves, respectively. This is followed by the conclusions.

## 2. Theory and Optimization Problem

### 2.1. Band Gap Analysis of Phononic Crystals

The governing equation of mechanic waves that propagate in a heterogeneous medium is given by:

(1) $$\rho (r)\ddot{u}=\nabla [\lambda (r)+2\mu (r)](\nabla \cdot u)-\nabla \times [\mu (r)\nabla \times u]$$

where *λ* and *μ* denote the Lame’s coefficients; *ρ* is the material density; $u={\left\{u_{x},u_{y},u_{z}\right\}}^{T}$ is the displacement vector, and r = (*x*, *y*) denotes the position vector. In this paper, we consider two dimensional phononic crystals with square lattice and assume the propagation of elastic waves is restricted to the *x*–*y* plane only. Noting that the wave field is independent of *z i.e.*, ∂u/∂z=0, Equation (1) can be split into two equations which govern the in-plane longitudinal and transverse waves (which is called in-plane wave mode), and one out-of-plane equation that governs the out-of-plane waves (which is called out-of-plane wave mode) as:

(2) $$\rho (r)\frac{\partial^{2}u_{x}}{\partial t^{2}}=\frac{\partial }{\partial x}\left[\left(\lambda \left(r\right)+2\mu \left(r\right)\right)\frac{\partial u_{x}}{\partial x}+\lambda \left(r\right)\frac{\partial u_{y}}{\partial y}\right]+\frac{\partial }{\partial y}\left[\mu \left(r\right)\left(\frac{\partial u_{x}}{\partial y}+\frac{\partial u_{y}}{\partial x}\right)\right]$$

(3) $$\rho (r)\frac{\partial^{2}u_{y}}{\partial t^{2}}=\frac{\partial }{\partial x}\left[\mu \left(r\right)\left(\frac{\partial u_{x}}{\partial y}+\frac{\partial u_{y}}{\partial x}\right)\right]+\frac{\partial }{\partial y}\left[\lambda \left(r\right)\frac{\partial u_{x}}{\partial x}+\left(\lambda \left(r\right)+2\mu \left(r\right)\right)\frac{\partial u_{y}}{\partial y}\right]$$

(4) $$\rho (r)\frac{\partial^{2}u_{z}}{\partial t^{2}}=\frac{\partial }{\partial x}\left[\mu \left(r\right)\frac{\partial u_{z}}{\partial x}\right]+\frac{\partial }{\partial y}\left[\mu \left(r\right)\frac{\partial u_{z}}{\partial y}\right]$$

**Figure 1:**
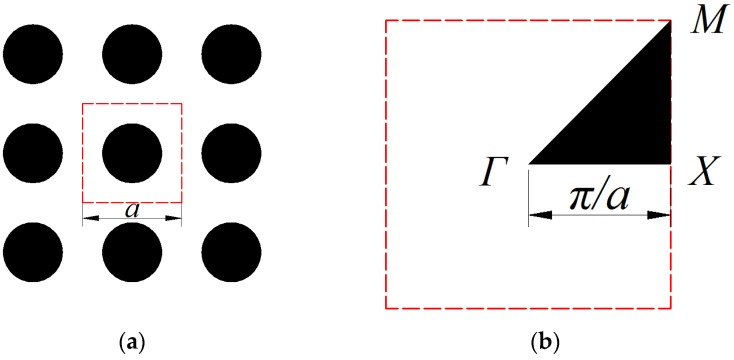
(**a**) Phononic crystals with 3 × 3 unit cells; and (**b**) irreducible first Brillouin zone (*Γ-Χ-Μ-Γ*).

According to Bloch’s theorem, for periodic structures the displacement vector **u**(**r**) should satisfy the form:

(5) $$u\left(r,k\right)=u_{k}\left(r\right)e^{i\left(kr\right)}$$

where **u***_k_*(**r**) is a periodic function of **r** with the same periodicity to the structure. **K** *=* (*k_x_*, *k_y_*) is the Bloch wave vector [21] By inserting Equation (5) into either Equations (2) and (3) or Equation (4), the governing equations can be converted to two eigenvalue problems for in-plane and out-of-plane waves, respectively. We could easily solve the problem by the finite-element method and both eigenvalue problems can be written as:

(6) $$\left(K\left(k\right)-\omega {\left(k\right)}^{2}M\right)u=0$$

where eigenvectors **u** *=* **u***_k_*(**r**). **K** and **M** are the stiffness matrix and mass matrix, respectively. It should be noted that, due to the symmetry of the unit cell, the above eigenvalue equations only need to be solved within the first Brillouin zone. Moreover, it has been verified that the searching area can be further reduced to the wave vectors on the boundary of the irreducible Brillouin zone for the calculation of band structures [22]. For a 2D phononic crystal with the square lattice shown in Figure 1a, the boundary of the irreducible first Brillouin zone is sides of the triangle *Γ-X-M-Γ* shown in Figure 1b. In the following numerical examples, the wave vectors **k** = (*k*_x_, *k*_y_) are appointed with 11 equally-spaced points along each boundary and start from the point *Γ* (0, 0), to *Χ*(π/*a*, 0), then *Μ*(π/*a*, π/*a*), and finally come back to the point *Γ*.

### 2.2. Static Effective Stiffness of Phononic Crystals

When cellular phononic crystals are considered as a component material for a device, the periodic unit cell would be very small compared with the size of the structural body. Therefore, the static effective elasticity tensor of cellular phononic crystals can be calculated by homogenization theory [23,24] in terms of the material distribution in the unit cell, which is stated as:

(7) $$E_{ij}^{H}=\frac{1}{\left|Y\right|}\int_{\Omega }{\left(\left\{\varepsilon_{0}^{i}\right\}-\left\{\varepsilon^{i}\right\}\right)}^{T}\left[E\right]\left(\left\{\varepsilon_{0}^{j}\right\}-\left\{\varepsilon^{j}\right\}\right)d\Omega$$

where $E_{ij}^{H}$ is homogenized elasticity tensor, *[**E**]* is the constitutive matrix at a given point, |Y| denotes the area of the unit cell Ω, *i*, *j* = 1, 2, 3 for two dimensional inhomogeneous structures, $\left\{\varepsilon_{0}^{i}\right\}$ are three linear independent test strain fields as $\left\{\varepsilon_{0}^{1}\right\}=\left\{1,0,0\right\}$, $\left\{\varepsilon_{0}^{2}\right\}=\left\{0,1,0\right\}$, $\left\{\varepsilon_{0}^{3}\right\}=\left\{0,0,1\right\}$, $\left\{\varepsilon^{i}\right\}$ are the introduced strain fields, which are the solutions to the standard finite element equation and subject to periodic boundary condition and the test strain fields $\left\{\varepsilon_{0}^{i}\right\}$.

The static stiffness of cellular structures is usually described by bulk modulus *κ^H^* or shear modulus *G^H^* and can be expressed in terms of the components of effective elasticity tensor $E_{ij}^{H}$ as:

(8) $$\kappa^{H}=\frac{1}{4}\left(E_{11}^{H}+E_{12}^{H}+E_{21}^{H}+E_{22}^{H}\right)$$

(9) $$G^{H}=E_{33}^{H}$$

To design a stiffer cellular phononic band gap crystal, the best way is to maximize bulk or shear modulus and the band gaps simultaneously. However, our numerical experiences show that it is impossible to achieve two goals at the same time, since the optimal solutions to maximize bulk or shear modulus and maximize phononic band gaps are in two opposite directions. An alternate way is to make a compromise by adding a bulk or shear modulus constraint to the optimization problem of maximizing band gaps, so that the resulting structures can possess a relatively big band gap while also maintaining a higher stiffness. In the following examples, dimensionless stiffness constraints are used instead of effective bulk or shear modulus. Specifically, *κ = κ^H^/κ_0_* and *G = G^H^/G_0_* are used as static effective bulk and shear modulus constraints, where *κ_0_* and *G_0_* are the bulk and shear moduli, respectively, of the solid material.

For a cellular structure with a volume fraction *V_f_* of solid material in the design domain, the maximum value of bulk or shear modulus should satisfy the Hashin–Shtrikman bounds for two-phase materials [25]. The corresponding dimensionless upper bounds of stiffness are given as below:

(10) $$\kappa_{upper}=\frac{V_{f}G_{0}}{\left(1-V_{f}\right)\kappa_{0}+G_{0}}$$

(11) $$G_{upper}=\frac{V_{f}\kappa_{0}}{\left(1-V_{f}\right)\left(\kappa_{0}+2G_{0}\right)+\kappa_{0}}$$

In the optimization of cellular phononic crystals, the stiffness constraint value should be not greater than *κ_upper_* and *G_upper_*, otherwise the optimization will tend to maximize the bulk or shear modulus other than band gaps. Thus, the ratio of *κ*/*κ_upper_* and *G*/*G_upper_* should be carefully chosen as the stiffness constraint. We will discuss this later in the numerical examples.

### 2.3. Optimization Problem

The goal of this paper is to design cellular phononic crystals with a desirable band gap by properly rearranging the distribution of air holes and solid material subject to a given material volume and certain stiffness. Considering the absence of the essential length scale in the governing equations, here we choose to optimize the relative band gap size, which is characterized by the gap-midgap ratio. To optimize the relative band gap size between the *n^th^* and (*n* *+* 1)*^th^* band for in-plane waves or out-of-plane waves, the optimization problem can be mathematically formulated with objective and constraint functions as follows:

(12) $$\mathrm{Maximize}:\;f\left(x_{e}\right)=2\frac{\min\omega_{n+1}\left(k\right)-\max\omega_{n}\left(k\right)}{\min\omega_{n+1}\left(k\right)+\max\omega_{n}\left(k\right)}$$

(13) $$\text{Subject to}:\;\begin{array}{ccc} \kappa \geq \kappa^{*}=\beta \kappa_{upper} & or & G\geq G^{*}=\beta G_{upper} \end{array}$$

(14) $$\begin{array}{cc} V_{f}^{*}=\sum_{e=1}^{N}x_{e}V_{e} & x_{e}=x_{\min}\mathrm{or}1 \end{array}$$

where the objective function *f*(*x_e_*) denotes the gap-midgap ratio between the *n^th^* and (*n* *+* 1)*^th^* bands which is defined by the percentage in the following band diagrams; ω*_n_*, ω*_n_*_+1_ are eigenfrequecies at target bands; κ^*^ and G^*^ are static effective bulk and shear modulus constraint, and *β* is the ratio of stiffness constraint over its upper bound value at a same volume. Obviously the magnitude of *β* is located in the range between 0 and 1; $V_{f}^{*}$ is the volume constraint; *x_e_* is the artificial design variable, which denotes the material type (air or solid material) for each element.

### 2.4. Bi-Directional Evolutionary Structural Optimization (BESO)

Here, we employ the bi-directional evolutionary structural optimization (BESO) method, a very efficient and effective topology algorithm in optimum material distribution problems for continuum structures, to resolve the optimization problem defined by Equations (12)–(14). BESO is a further developed version of evolutionary structural optimization (ESO), which was originally proposed by Xie and Steven [26,27] in the early 1990s. The basic concept of ESO is to gradually remove low efficient materials from the structure so that the rest part evolves to an optimum. BESO allows adding materials to the most efficient regions as well as deleting insufficient ones [28,29]. In the present paper, we use the new BESO method which is proposed by Huang and Xie and greatly improved the robustness and computational efficiency of the original ESO and BESO methods [30,31,32]. It has also been demonstrated that the current BESO method is well capable of the design of periodic microstructures for cellular materials and composites, such as the photonic crystals [33,34,35,36].

**Figure 2:**
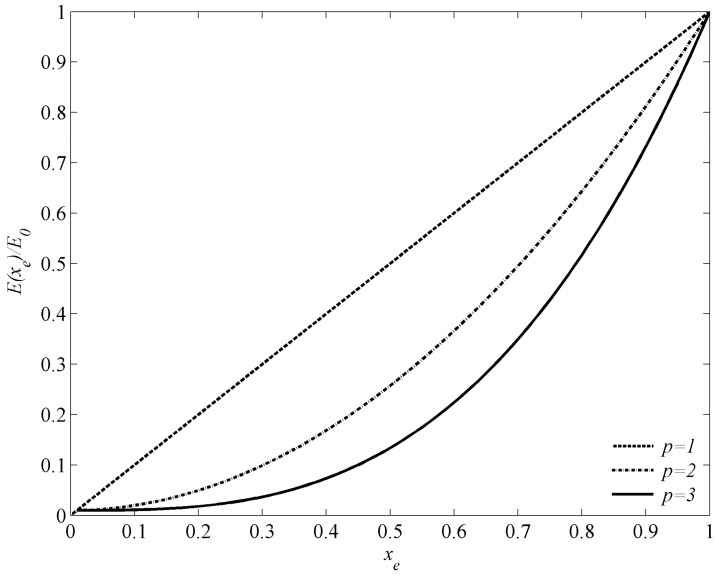
Material interpolation scheme with *x_min_* = 0.01.

The problem of maximizing the band gap size for phononic crystals is in essence how to arrange the spatial distributions of the solid material and air within the unit cell. In other words, the topology optimization problem can be transferred to alter the artificial design variable of each element after the finite element discretization. In order to get the gradient information of object function with respect to the design variables, it is necessary to interpolate the material properties between air and the solid material. In the optimization of composite phononic crystals, which consisted of two solid materials, a simple linear material interpolation scheme works very well [13]. However, artificial localized modes are often encountered in low density regions for the case of optimization of cellular phononic crystals due to the extremely high contrast of mass and stiffness between air and the solid material. To avoid this problem, we apply a similar material interpolation scheme with penalization as in the studies on the topology optimization of continuum structures for natural frequencies [37]. The interpolation scheme is given as:

(15) $$\rho \left(x_{e}\right)=x_{e}\rho_{0}$$

(16) $$E\left(x_{e}\right)=\left[\frac{x_{\min}-x_{\min}^{p}}{1-x_{\min}^{p}}\left(1-x_{e}^{p}\right)+x_{e}^{p}\right]E_{0}\;\begin{array}{c} \left(0<x_{\min}\leq x_{e}\leq 1\right) \end{array}$$

where *ρ*_0_ and *E*_0_ represent the density and Young’s modulus of solid material, respectively; *p* is the penalty exponent; *x_e_* stands for a design variable, *x_e_* = *x_min_* denotes element *e* is composed of air, and *x_e_* *=* 1 means element *e* is composed of solid material. To avoid singularity in finite element analysis, *x_min_* in the calculation is usually set to be a very small value that is slightly larger than 0. In the following example, the value is chosen as *x_min_* = 1 × 10^−6^. Current material interpolation scheme for different *p* with *x_min_* = 0.01 is plotted in Figure 2. It is clearly seen that when *x_e_* approaches the ends 0 or 1, the current model is same as the linear material interpolation case.

It should be pointed out that the discrete design variable *x_e_* is set to be *x*_min_ or 1 only in the traditional BESO method [32,37]. However, the optimization of phononic crystals is very sensitive to the variation of design variable, so the change of design variable in each iteration is limited to be Δ*x_e_* = 0.1 to stabilize the optimization process. In other words, *x_e_* can take 11 discrete values, as $x_{e}\in \left\{x_{\min},0.1,\cdots ,0.9,1\right\}$. Our numerical experience in the optimization of composite case shows that the discrete scheme could also lead to a clear solid/void final topology.

## 3. Numerical Implementation and BESO Procedure

### 3.1. Reformulation of Objective Function

Current optimization problem stated in Equations (12)–(14) has multiple constraints, including a stiffness constraint and a volume constraint. Like the optimization problem of continuum structures with an additional displacement constraint using ESO method [38], the stiffness constraint can be added by introducing a Lagrangian multiplier. The objective function can be modified for the bulk modulus constraint to:

(17) $$\mathrm{Maximize}:\;f\left(x_{e}\right)=2\frac{\min\omega_{n+1}\left(k\right)-\max\omega_{n}\left(k\right)}{\min\omega_{n+1}\left(k\right)+\max\omega_{n}\left(k\right)}+\lambda \left(\kappa -\kappa^{*}\right)$$

and for the shear modulus constraint to:

(18) $$\mathrm{Or}:\;f\left(x_{e}\right)=2\frac{\min\omega_{n+1}\left(k\right)-\max\omega_{n}\left(k\right)}{\min\omega_{n+1}\left(k\right)+\max\omega_{n}\left(k\right)}+\lambda \left(G-G^{*}\right)$$

When the bulk or shear modulus is equal to the prescribed ones, the above equations are equivalent to the original objective function defined in Equation (13). Otherwise, taking the bulk modulus constraint case for example, if *κ* > κ^*^, which means the constraint is already satisfied and λ is set to be 0; if *κ* < κ^*^, which means the constraint is not satisfied yet and we need to maximize the bulk modulus first, so λ needs to be infinity. The case of shear modulus constraint is the same as that of bulk modulus. The determination of Lagrangian multiplier will be discussed later. After this modification, optimization of cellular phononic crystals with stiffness constraint can be solved as the standard band gap optimization problem.

### 3.2. Sensitivity Analysis

The gradient of objective function with respect to the change of design variable *x_e_* defined in Equation (17) for bulk modulus constraint case can be calculated by:

(19) $$\alpha =\frac{\partial f(x_{e})}{\partial x_{e}}=4\frac{\max\omega_{n}\left(k\right)\frac{\partial \min\omega_{n+1}\left(k\right)}{\partial x_{e}}-\min\omega_{n+1}\left(k\right)\frac{\partial \max\omega_{n}\left(k\right)}{\partial x_{e}}}{{\left(\min\omega_{n+1}\left(k\right)+\max\omega_{n}\left(k\right)\right)}^{2}}+\lambda \frac{\partial \kappa }{\partial x_{e}}$$

and for shear modulus constraint case by:

(20) $$\alpha =\frac{\partial f(x_{e})}{\partial x_{e}}=4\frac{\max\omega_{n}\left(k\right)\frac{\partial \min\omega_{n+1}\left(k\right)}{\partial x_{e}}-\min\omega_{n+1}\left(k\right)\frac{\partial \max\omega_{n}\left(k\right)}{\partial x_{e}}}{{\left(\min\omega_{n+1}\left(k\right)+\max\omega_{n}\left(k\right)\right)}^{2}}+\lambda \frac{\partial G}{\partial x_{e}}$$

where the sensitivities of eigenfrequencies can be expressed by the following equation with the assumption that eigenvectors are nomalized to the global mass matrix for a given wave vector **k**:

(21) $$\frac{\partial \omega_{n}}{\partial x_{e}}=\frac{1}{2\omega_{n}}u{\left(k\right)}_{n}^{T}\left(\frac{\partial K}{\partial x_{e}}-\omega_{n}^{2}\frac{\partial M}{\partial x_{e}}\right)u{\left(k\right)}_{n}$$

where **u**(**k**)*_n_* is the corresponding eigenvector; **K** and **M** are the elemental stiffness and mass matrix, respectively.

According to the relations in Equations (8) and (9), the sensitivities of homogenized bulk or shear modulus can be computed as:

(22) $$\frac{\partial \kappa^{H}}{\partial x_{e}}=\frac{1}{4}\left(\frac{\partial E_{11}^{H}}{\partial x_{e}}+\frac{\partial E_{12}^{H}}{\partial x_{e}}+\frac{\partial E_{21}^{H}}{\partial x_{e}}+\frac{\partial E_{22}^{H}}{\partial x_{e}}\right)$$

(23) $$\frac{\partial G^{H}}{\partial x_{e}}=\frac{\partial E_{33}^{H}}{\partial x_{e}}$$

Using the adjoint varibale method [39], the sensitivity of the homogenized elasticity tensor with respect to design variable can be derived as:

(24) $$\frac{\partial E_{ij}^{H}}{\partial x_{e}}=\frac{1}{\left|Y\right|}\int_{\Omega }{\left(\left\{\varepsilon_{0}^{i}\right\}-\left\{\varepsilon^{i}\right\}\right)}^{T}\frac{\partial E_{ij}}{\partial x_{e}}\left(\left\{\varepsilon_{0}^{j}\right\}-\left\{\varepsilon^{j}\right\}\right)d\Omega$$

Considering the material interpolation scheme defined in Equation (16), the sensitivity of the homogenized elasticity tensor can be written as:

(25) $$\frac{\partial E_{ij}^{H}}{\partial x_{e}}=\frac{px_{e}^{p-1}}{\left|Y\right|}\frac{1-x_{\min}}{1-x_{\min}^{p}}\int_{\Omega }{\left(\left\{\varepsilon_{0}^{i}\right\}-\left\{\varepsilon^{i}\right\}\right)}^{T}E_{ij}^{0}\left(\left\{\varepsilon_{0}^{j}\right\}-\left\{\varepsilon^{j}\right\}\right)d\Omega$$

where $E_{ij}^{0}$ is the elasticity tensor of solid material.

Up to now, the only unknown parameter in Equation (19) or Equation (20) is the Lagrangian multiplier, which highly influences the relative ranking of the overall sensitivity of the objective function.

### 3.3. Determination of the Lagrangian Multiplier

Before the calculation of the overall sensitivity of the objective function, the Lagrangian multiplier λ should be determined first. Here we introduce an intermediate parameter *w* in our program, which is defined by:

(26) $$\lambda =\frac{1-w}{w}$$

where *w* is a constant value which can vary from a extremely small value *w*_min_ e.g., 10^−20^, to 1, so the resulting Lagrangian multiplier λ can be located in the range of 0 to infinity.

Initially, the search for the proper value of *w* starts from *w* = 1 with a lower bound *w*_lower_ = *w*_min_ = 10^−20^ and an upper bound *w*_upper_ = *w*_max_ = 1. Then, with the Lagrangian multiplier computed by Equation (26), the sensitivity of objective function is calculated by Equation (19) or Equation (20). Then, based on the ranking of the sensitivity the design variables are updated to satisfy the volume fraction in the next iteration. The bulk or shear modulus in the next iteration can be approximately estimated by the variation of bulk or shear modulus and the value in the current iteration, *κ^i^* or *G^i^*, as:

(27) $$\kappa^{i+1}\approx \kappa^{i}+\sum_{e}\frac{d\kappa }{dx_{e}}\Delta x_{e}$$

or:

(28) $$G^{i+1}\approx G^{i}+\sum_{e}\frac{dG}{dx_{e}}\Delta x_{e}$$

where $\frac{d\kappa }{dx_{e}}$ and $\frac{dG}{dx_{e}}$ are computed by Equations (22) and (23). After that, the estimated bulk or shear modulus will be compared with the current bulk or shear modulus constraint. If *κ^i+^*^1^ < κ^*^, which means the current constraint is not satisfied and current Lagrangian multiplier λ is too small, we would update *w* with a smaller value by:

(29) $$\hat{w}=\frac{w+w_{\mathrm{lower}}}{2}$$

Meanwhile, the upper bound of *w*_upper_ will be moved to *w*. If *κ^i+^*^1^ > κ^*^, which means the current constraint is already satisfied, still we would update *w*, however, with a larger value by:

(30) $$\hat{w}=\frac{w+w_{\mathrm{upper}}}{2}$$

and the lower bound *w*_lower_ will be moved to *w*. The Lagrangian multiplier is updated at the same time. This procedure will be repeated until the difference between *w*_upper_ and *w*_lower_ is less than 10^−15^. Finally the appropriate Lagrangian multiplier is obtained after several iterations.

### 3.4. BESO Procedure

In the optimization of composite phononic band gap crystals, our numerical experiences show that the filter scheme will lead to more reliable and steady evolution history. Therefore, we continue to apply a filter by averaging the elemental sensitivity with its neighbor elements in the optimization of cellular phononic crystals. Instead of averaging the overall sensitivity calculated by the Equation (19) or Equation (20), the filter is separately applied to the sensitivity of gap-midgap ratio and homogenized bulk or shear modulus since our numerical simulation indicates that filtering the sensitivity separately could better avoid the checkerboard problem [40]. The filter scheme refers to [37].

**Figure 3:**
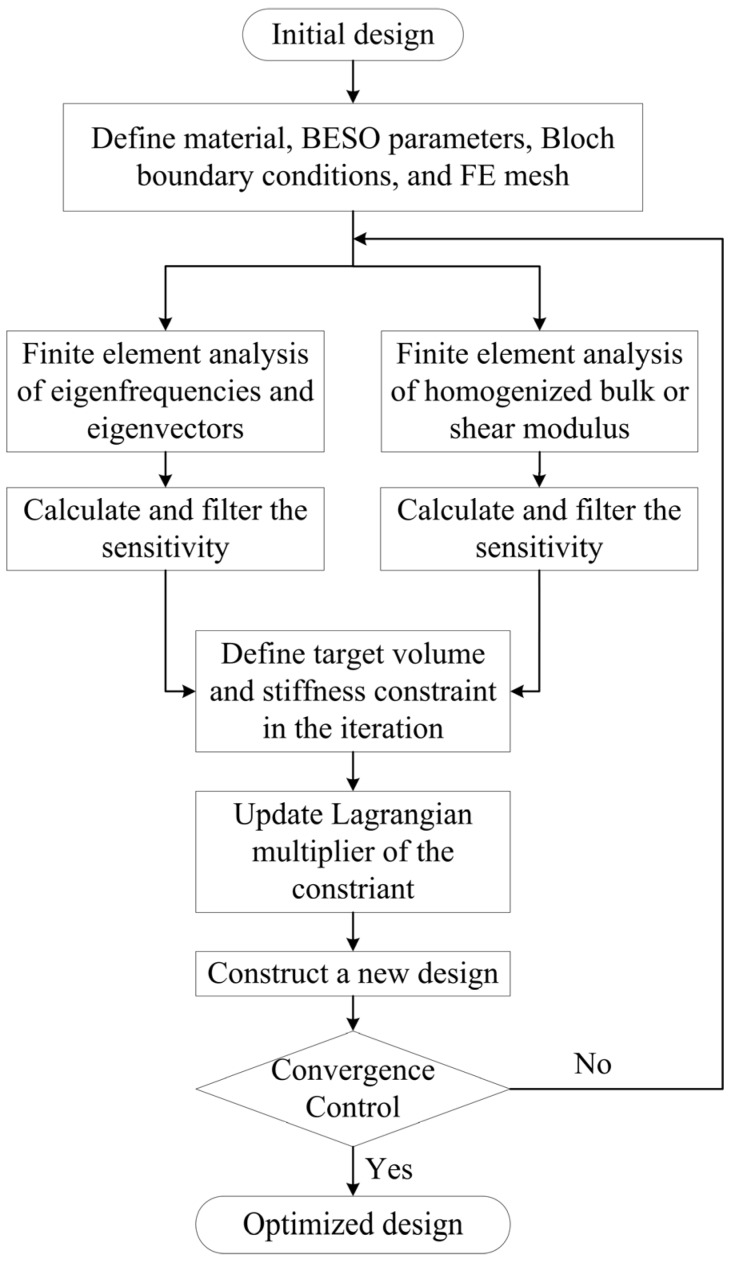
Flow chart of optimization procedure using BESO method.

The detailed optimization procedure using BESO method can be illustrated by the Figure 3. We choose a simple or random unit cell as the initial design and discretize the design domain by a finite element mesh. Then we define the objective volume fraction of the solid material $V_{f}^{*}$, evolutionary ratio *ER* and penalty *p* in the material interpolation scheme. In the following step, we first conduct finite element analysis for several discrete wave vectors **k** along the first Brillouin zone with Bloch boundary conditions to get corresponding eigenvalues and eigenvectors to Equation (6). After this, the sensitivities of gap-midgap ratio for each element are calculated and filtered. Then we use homogenization method to get the effective bulk or shear modulus (*κ^i^* or *G^i^*) of current configuration. Additionally, the sensitivities of homogenized parameters are calculated and filtered. Before the update of the Lagrangian multiplier of the constraint, the target volume in the next iteration is defined as:

(31) $$\begin{array}{cc} V_{f}^{i+1}=V_{f}^{i}\left(1-ER\right) & \begin{array}{cc} when & V_{f}^{i}>V_{f}^{*} \end{array} \end{array}$$

where *i* denotes the iteration number. Once the final volume fraction $V_{f}^{*}$ is achieved, the volume will be kept as a constant. At the same time, corresponding bulk modulus constraint κ^*^ or shear modulus constraint G^*^ at the target volume fraction in next iteration is computed by Equation (10) or Equations (11) and (13) with a given constraint ratio *β*. *ER* = 2%, $V_{f}^{*}$ = 50% and *β* = 0.3 are used throughout this paper, unless otherwise stated. Thereafter, the appropriate Lagrangian multiplier in the next iteration is determined by the procedure illustrated in previous section. Then the overall sensitivity of the objective function can finally be obtained.

Based on the relative ranking of the calculated overall sensitivity, a threshold of the sensitivity number, $\alpha_{th}^{i}$, is determined by using the bi-section method so that the volume fraction in the next iteration is equal to $V_{f}^{i+1}$. The design variable of each element is modified by comparing its sensitivity number $\alpha_{e}^{i}$ with the threshold as:

(32) $$x_{e}^{i+1}=\left\{ \begin{array}{l} \min(x_{e}^{i}+\Delta x,1),\;\mathrm{if}\;\alpha_{e}^{i}>\alpha_{th}^{i}\\ \max(x_{e}^{i}-\Delta x,0),\;\mathrm{if}\;\alpha_{e}^{i}<\alpha_{th}^{i} \end{array}\right.$$

where Δx=0.1 throughout the paper, which means BESO uses discrete design variables.

The above procedure is repeated until the convergence criteria are satisfied. It should be pointed out that the stiffness constraint in the optimization process is not a constant value like volume constraint, and would evolve with the volume fraction for the sake of stability of the evolutionary history. Additionally, although discrete intermediate design variables are used during the optimization process, the optimized design we obtained can naturally converge to an almost solid/void design due to the adoption of the material interpolation scheme.

## 4. Results and Discussion

To demonstrate the capability of the proposed algorithm, this section will present a number of band gap optimization results we obtained for the out-of-plane mode, in-plane mode, and the combined mode of these two with bulk and shear modulus constraints, respectively. In the following examples, we consider the design of a 2D cellular phononic crystal with square lattice. The objective is to maximize the relative band gap size between two adjacent bands. All the numerical simulations are conducted with a volume constraint $V_{f}^{*}$ = 50%.

Silicon is used as the solid material due to its popularity in the design of photonic crystals, which are the electromagnetic analog of phononic crystals and have been extensively studied. The physical properties of silicon are given as *ρ* = 2330 kg/m^3^, *λ* = 85.502 GPa, and *μ* = 72.835 GPa [17]. The unit cell with dimensionless lattice length *a* = 1 is discretized into 64 × 64 linear four-node elements. All of the evolutionary rates of volume fraction used in the BESO procedure are *ER* = 2%. The filter radius for the sensitivity of band gap is r*_min1_* = $\sqrt{2}a/30$ while for the sensitivity of homogenized bulk or shear modulus is r*_min2_* = $\sqrt{2}a/40$. The eigenfrequencies (*ω*) in the band structures are normalized by *ωa/2πC*, where *C* = 340 m/s denotes the wave speed in air.

### 4.1. Out-of-Plane Mode

#### 4.1.1. Influence of Stiffness Constraint

**Figure 4:**
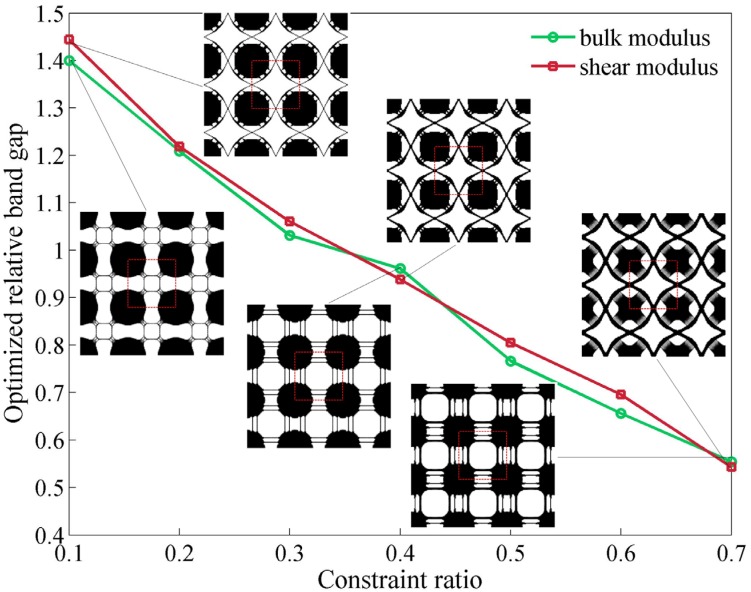
Optimized relative band gap against different constraint ratio with bulk and shear modulus constraints.

As mentioned in Section 2.2, the value of stiffness constraint directly affects the final optimization results of cellular phononic band gap crystals. To investigate the influence of different bulk and shear modulus constraint on the optimized band gap size, we conduct a series of optimizations starting from a same initial design with the constraint ratio *β* defined in Equation (13), changing from 0.1 to 0.7. The relations of optimized relative band gaps and constraint ratio *β* are plotted in Figure 4 along with the corresponding final topologies consisting of 3 × 3 unit cells. The optimized unit cell is shown in the red dashed box. It can be clearly seen that the optimal relative band gaps size for both bulk and shear modulus constraint case declines continuously with the increase of constraint ratio while the shapes inside the unit cell does not vary a lot. The final relative band gap sizes with shear modulus constraint are slightly bigger than those with bulk modulus constraint but the differences are quite small compared with their net value.

The maximum relative band gap size we obtained is up to 144.41% with the shear modulus constraint as *β_G_* = 0.1, which breaks the record value in the literature [16,17]. This number decreases dramatically by around 90% to 54.27% when the shear modulus constraint ratio *β_G_* increases from 0.1 to 0.7. Meanwhile the corresponding topology with smaller shear modulus constraint tends to have many tiny and slim connections, and these connections gradually disappear or become thicker when the constraint ratio *β_G_* is growing.

The situation for bulk modulus constraint case is very similar. The maximum relative band gap size with the bulk modulus constraint *β_κ_* = 0.1 is 140.02% while the number drops to 55.41% as the bulk modulus constraint increases to *β_κ_* = 0.7. Compared with the topologies we obtain with a shear modulus constraint, optimized structures in this group have different slim connections, while the main shapes of the solid material in the unit cell remain in similar positions.

Figure 4 indicates that the increase of stiffness constraint ratio will lead to a significant drop of optimized relative band gap size. As the bulk or shear modulus constraint gets bigger, the connections in the corresponding resulting topologies become thicker, which are favorable for future manufacture. To make a compromise between the band gap size and stiffness, the stiffness constraint ratio is set as *β_κ_* or *β_G_* = 0.3 for bulk and shear modulus constraint cases in the following examples.

**Figure 5:**
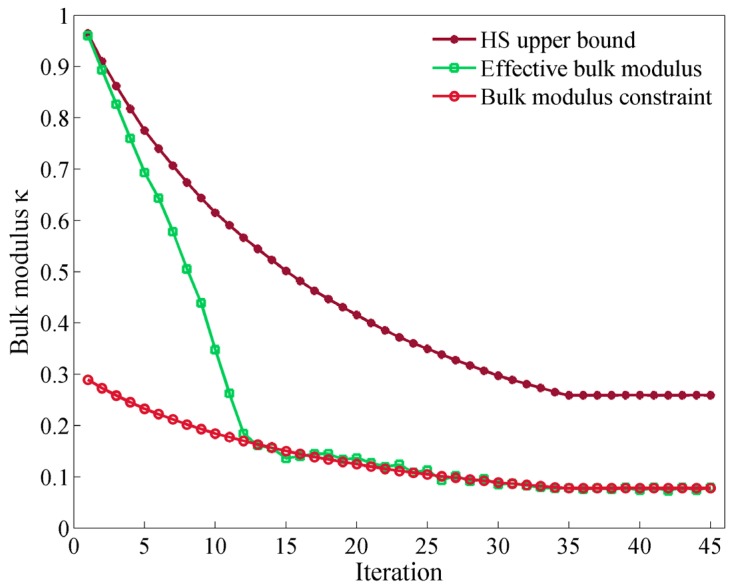
Evolution histories of the effective bulk modulus, bulk modulus constraint and corresponding HS upper bound in the optimization process with constraint ratio *β_κ_* = 0.3.

As mentioned above, we adopt an evolutionary stiffness constraint in the optimization process. As an illustration example, the evolution histories of effective bulk modulus and bulk modulus constraint in each iteration step and the corresponding HS upper bound are plotted in Figure 5 for the optimization case with constraint ratio *β_κ_* = 0.3. At the beginning, the effective bulk modulus is almost equal to the HS upper bound value, which means BESO starts from an initial configuration that is almost consisted of only solid materials. Based on the relation between the effective bulk modulus and the corresponding constraint value, the whole process can be divided into two stages. At the first stage, the bulk modulus constraints are far less than the effective bulk moduli calculated for the current configurations, indicating in these iterations the Lagrangian multiplier is equal to 0 and the objective is only to maximize the relative band gap size. It is observed that the increasing of the band gap in the first stage leads to a dramatic drop of the effective bulk modulus which, once again, reveals that the optimization trends for these two goals are opposite. At the second stage, the Lagrangian multiplier begins to play an important role in the balance of the band gap optimization and the effective bulk modulus optimization. It can be clearly seen that the effective bulk modulus keeps almost the same value as the bulk modulus constraint in the following optimization.

**Figure 6:**
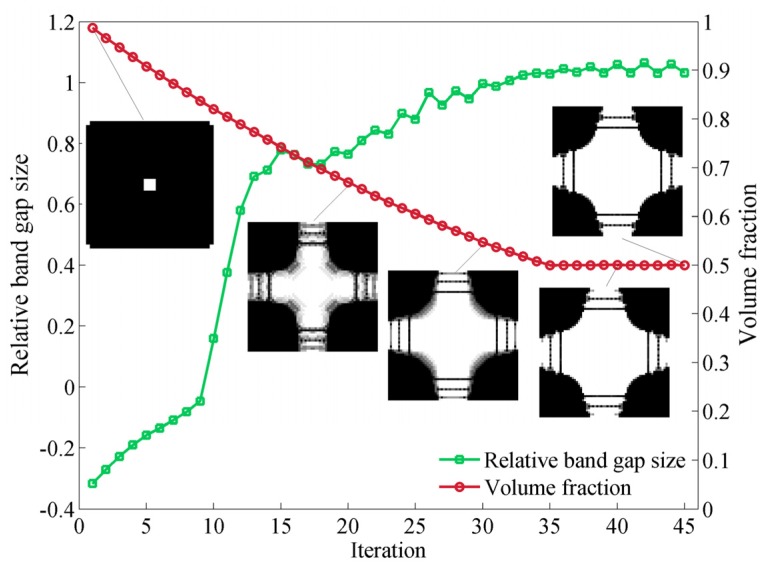
Evolution histories of the relative band gap size, volume fraction and topology in the optimization process with bulk modulus constraint *β_κ_* = 0.3.

The variation of the volume fraction, band gap size and topology of the unit cell for the same case during the whole optimization process is presented in Figure 6. We start from a simple unit cell, with centered and cornered holes, and the initial volume fraction *V_f_* is approximately equal to 1, which is in consistent with the initial effective bulk modulus. Initially the gap size is a negative value, indicating that there is no band gap between the first and second band. When the volume fraction steadily drops to the predefined constraint value 50%, the band gap size first gradually increases from a negative value to 0, and then rapidly rises after the opening of the band gap. When the Lagrangian multiplier begins to work, the band gap size experiences a fluctuating period and finally achieves its maximum value after the volume fraction is fixed. Meanwhile, the unit cell evolves to an optimal state with almost no grey elements. It takes less than 50 steps for this case to converge to an optimized structure. Furthermore, the optimized configuration is similar to the result reported in the literature [16].

#### 4.1.2. Out-of-Plane Results with Bulk Modulus Constraint *β_κ_* = 0.3

**Figure 7:**
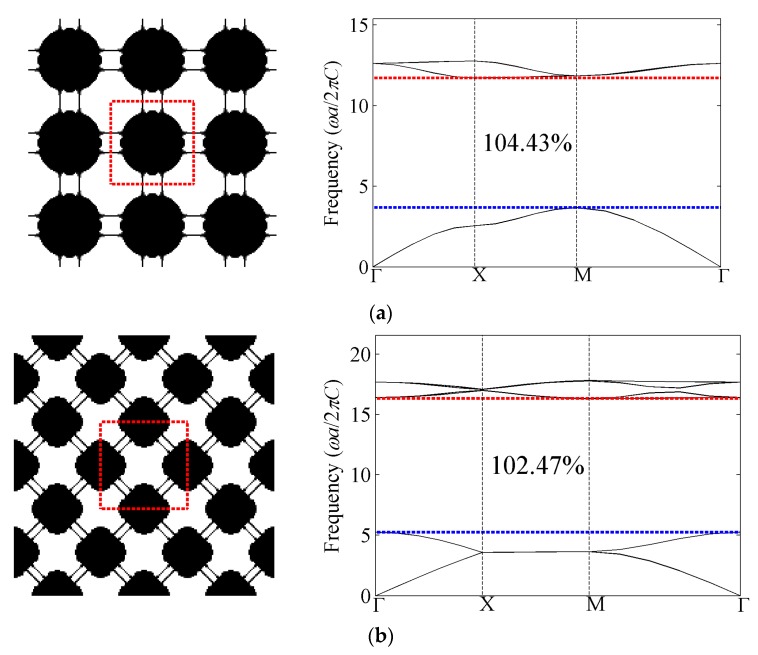

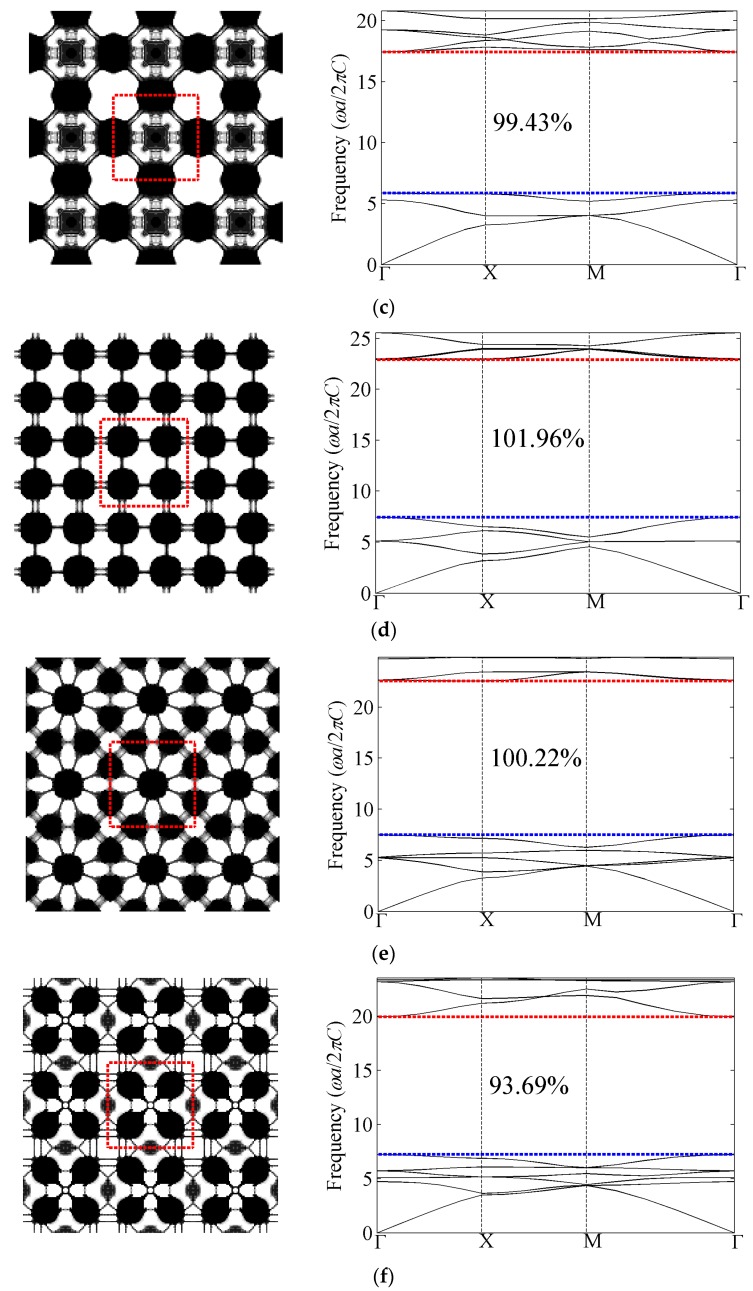

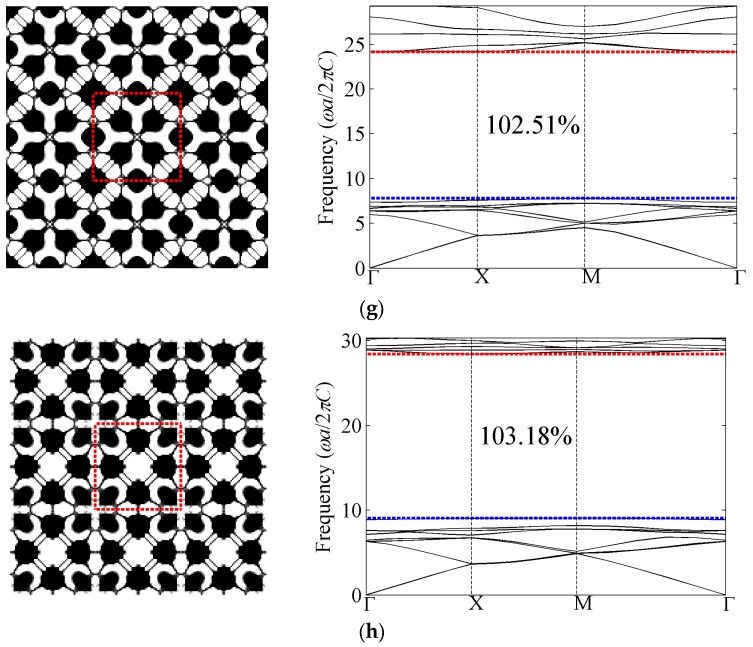
Optimized topologies and corresponding band structures for out-of-plane mode with bulk modulus constraint. The black and white colors represent silicon and air, respectively. (**a**) The first band gap; (**b**) the second band gap; (**c**) the third band gap; (**d**) the fourth band gap; (**e**) the fifth band gap; (**f**) the sixth band gap; (**g**) the seventh band gap; and (**h**) the eighth band gap.

All the optimized cellular phononic band gap crystals with bulk modulus constraint *β_κ_* = 0.3 and their corresponding band structures for the first eight band gaps of the out-of-plane modes are presented in Figure 7. The first band gap denotes the gap between the first and second band of the diagram, while the second band gap means the gap between the second and the third band of the diagram, and so on. As shown in the band structures, the optimized relative band gap sizes are around 100%. From the perspective of topologies, the complexity is increasing as the target band gap becomes higher. Compared with the optimization topologies of composite phononic crystals, which indicates the heavy and stiff material tends to be isolated by the light and soft material, the solid media also shows the trend to be isolated by air while still connected by thin structures. These slim connections are required so as to support the propagation of the shear components of mechanical waves; otherwise, only longitudinal waves could travel through the totally-isolated solid media. If those slim connections are removed, the remaining topologies are almost the same as those of composite phononic crystals [20].

Sigmund and Hougaard [41] have investigated the geometric properties of photonic crystals with maximum band gaps and found that the number of inclusions (for transverse electric modes) or subpartitions (for transverse magnetic modes) is interestingly equal to the band number at the lower edge of the band gap. It is also observed that the rule in TE modes of photonic crystals holds for the optimization results for out-of-plane mode of phononic crystals, except that the inclusions are linked to each other by slim connections.

#### 4.1.3. Out-of-Plane Results with Shear Modulus Constraint *β_G_* = 0.3

**Figure 8:**
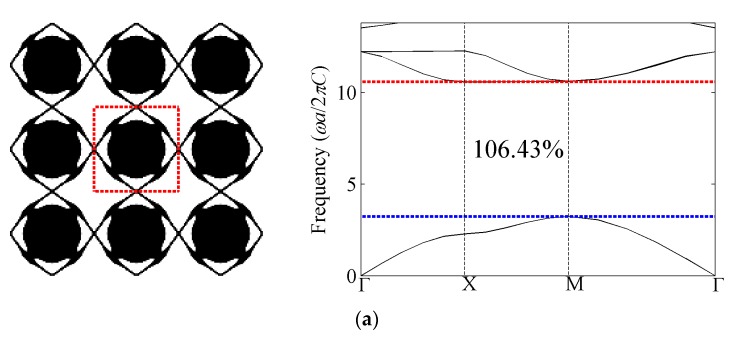

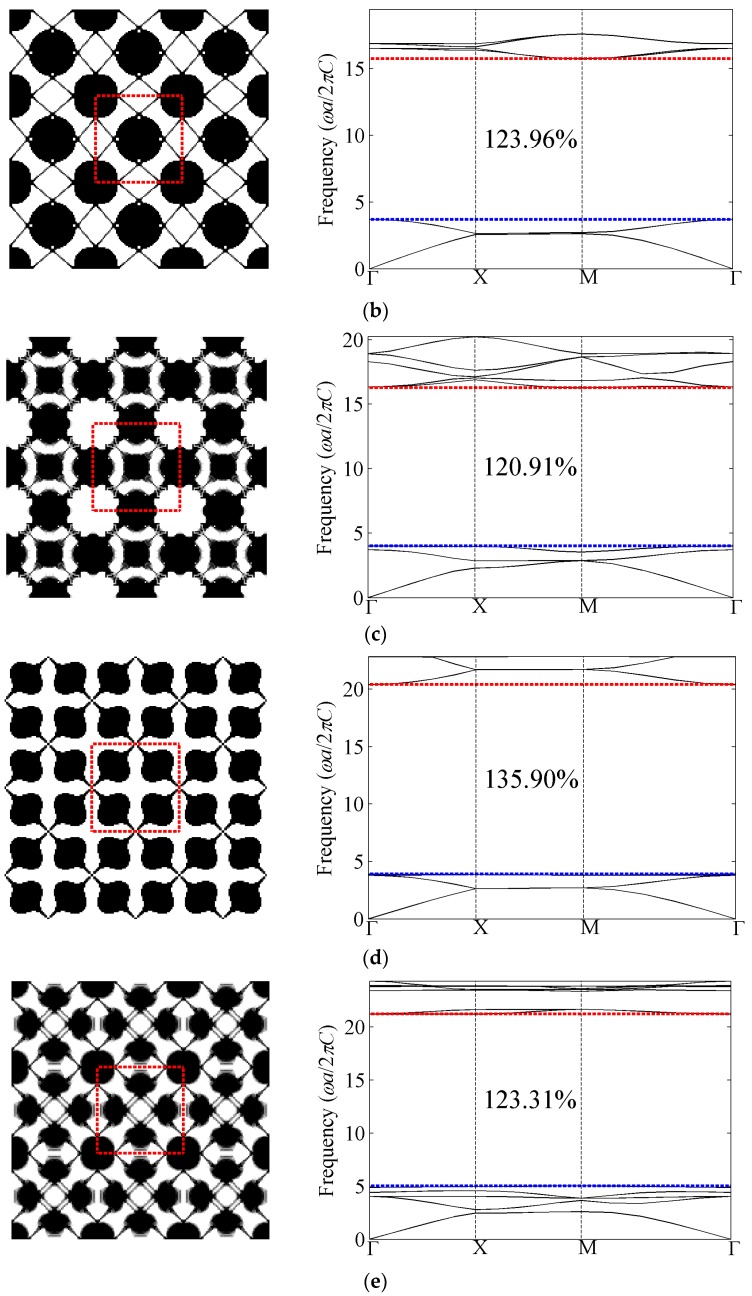

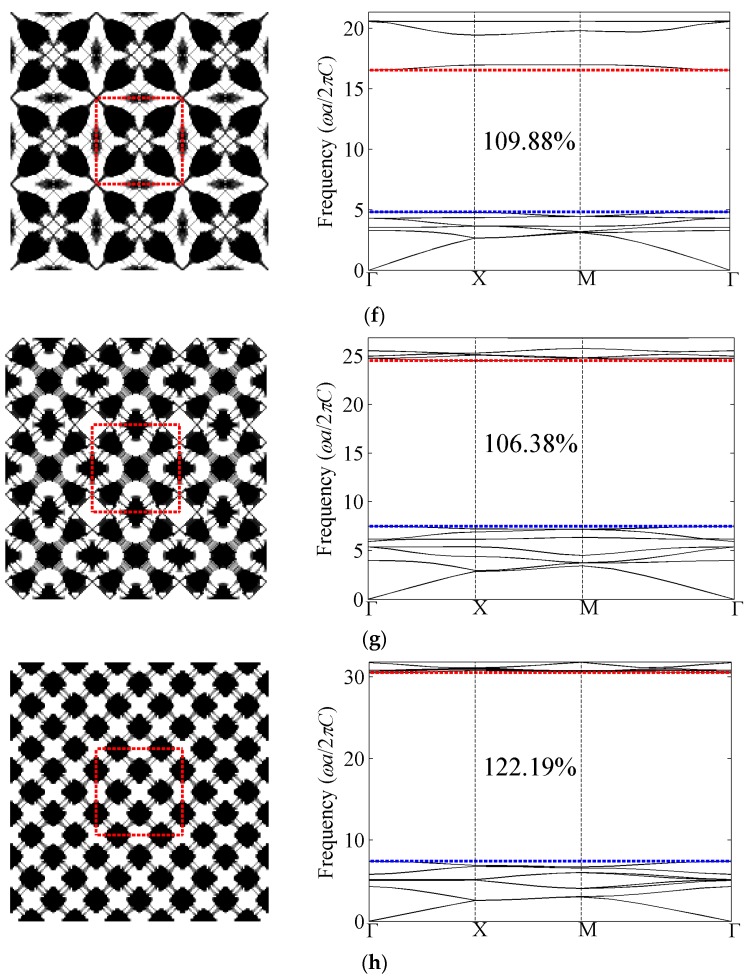
Optimized topologies and corresponding band structures for out-of-plane mode with shear modulus constraint. (**a**) The first band gap; (**b**) the second band gap; (**c**) the third band gap; (**d**) the fourth band gap; (**e**) the fifth band gap; (**f**) the sixth band gap; (**g**) the seventh band gap; and (**h**) the eighth band gap.

Figure 8 presents the optimized phononic band gap crystals with shear modulus constraint *β_G_* = 0.3 and their corresponding band structures for the first eight band gaps of the out-of-plane modes. The optimized relative band gap sizes with shear modulus constraint are all above 106%, which are larger than the bulk modulus constraint case. In addition, the normalized eigenfrequencies at the lower edge of the gap in the optimized structures are smaller than their counterpart cases with bulk modulus constraints, which indicates results with shear modulus constraints are more favorable for low-frequency-related applications. In terms of optimized topologies, the distributions of solid inclusions within the unit cell are basically akin to these with the bulk modulus constraint case. That is to say the number of the solid parts in the optimized topologies is still equal to the band number. However, the connections are different from those cases with bulk modulus constraint.

### 4.2. In-Plane Mode

It is more complex to optimize band gap for in plane mode of phononic crystals since both longitudinal and transverse polarizations exist in this mode. This is comparable to the optimization of a complete band gap between TE and TM mode in photonic crystals. Therefore, the resulting optimized structures are more complicated than those for out-of-plane mode.

#### 4.2.1. In-Plane Results with Bulk Modulus Constraint *β_κ_* = 0.3

**Figure 9:**
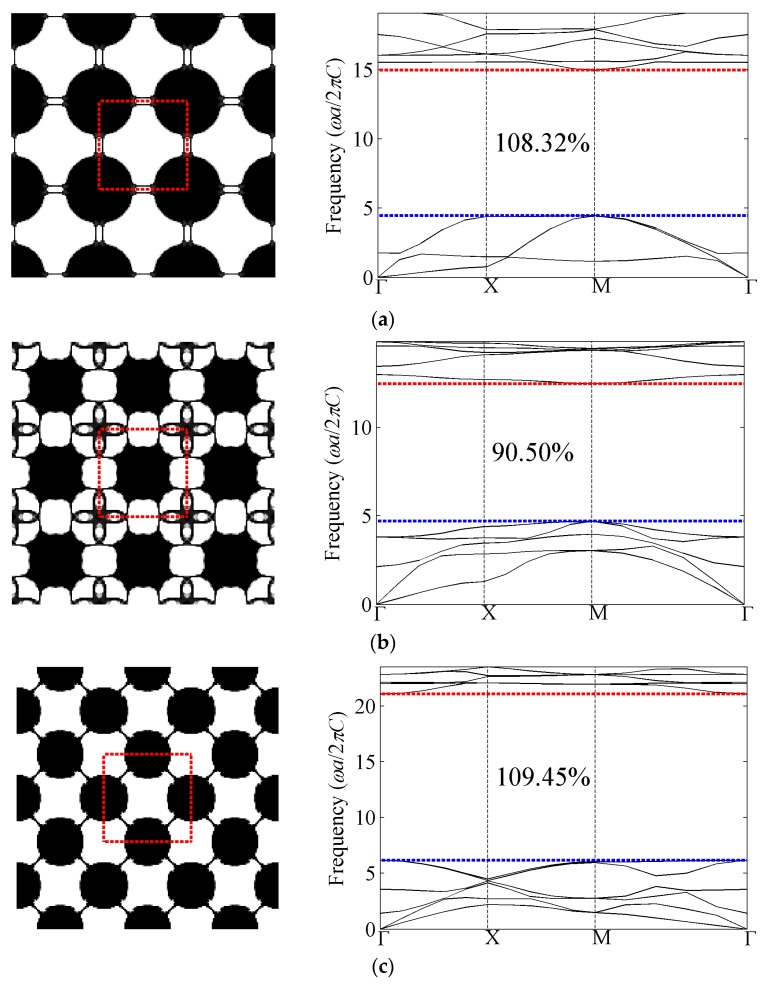
Optimized topologies and corresponding band structures for in-plane mode with bulk modulus constraint. (**a**) The third band gap; (**b**) the fifth band gap; and (**c**) the sixth band gap.

We successfully obtain three optimized topologies among the first six phononic band gap crystals for in plane waves with bulk modulus constraint *β_κ_* = 0.3 as shown in Figure 9. Compared with results for out-of-plane waves, the relative band gap sizes obtained for in-plane waves are smaller while the corresponding topologies are more complicated and have more delicate connections. For example, the optimized structure for fifth band gap has many complicated connections and almost no recognized solid circular or square areas. Moreover, there is no obvious pattern for the distributions of solid material in the optimized structures in this case. Among these results, the third and sixth phononic crystals with maximized band gap are relatively simple and similar to the first and second configurations for out-of-plane waves, respectively.

#### 4.2.2. In-Plane Results with Shear Modulus Constraint *β_G_* = 0.3

**Figure 10:**
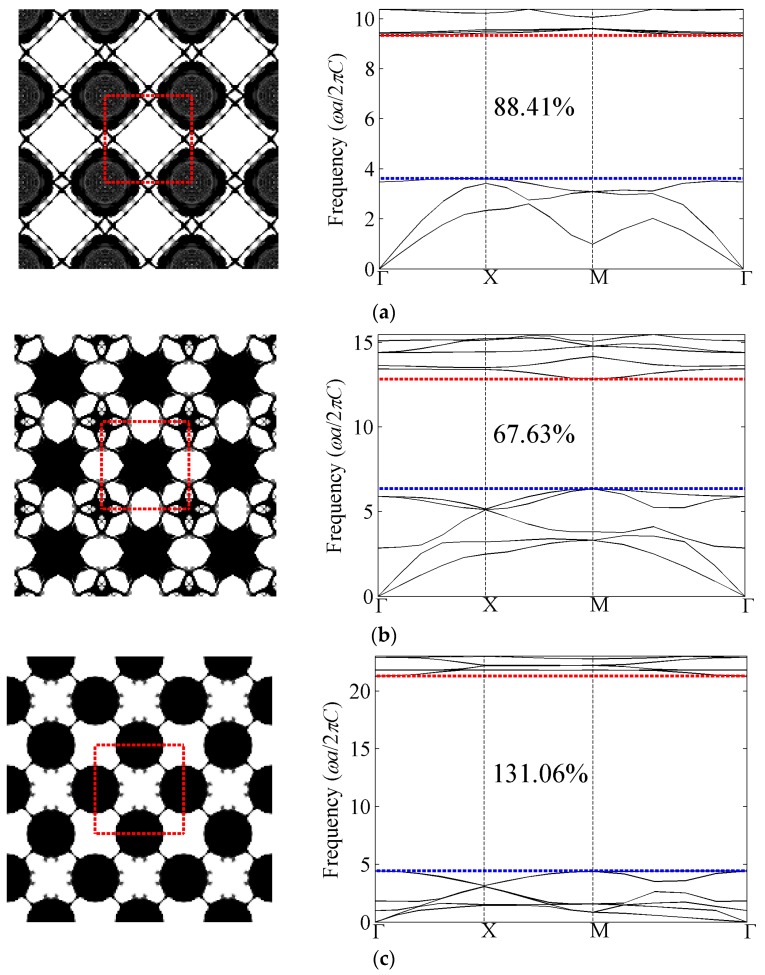
Optimized topologies and corresponding band structures for in-plane mode with shear modulus constraint. (**a**) The third band gap; (**b**) the fifth band gap; and (**c**) the sixth band gap.

Optimization results with shear modulus constraint *β_G_* = 0.3 for in-plane waves are presented in Figure 10. Different from the cases of out-of-plane waves, optimized relative band gap sizes for in plane waves with shear modulus constraint are slightly smaller than that with bulk modulus constraint.

Compared with results with bulk modulus constraint, the optimized topologies are similar. It is worthy to mention that a slight variation of connections could lead to dramatic change of the outcome band gap size for instance the sixth band gap. Double-checking the resulting structures with both constraints is recommended in practical application to ensure a larger band gap size.

## 5. Conclusions

This paper has systematically investigated the optimized topological design of phononic band gap crystals subject to a volume constraint and a bulk or shear modulus constraint, respectively. The BESO algorithm has been used to seek the optimal distribution of solid material within the square unit cell. The static effective bulk or shear modulus is calculated by a homogenization theory to ensure that the stiffness constraint is satisfied in spite of the variation of the topology in the optimization process. Various optimization results are presented for out-of-plane and in-plane mode and the effects of the stiffness constraint on the topological design of the cellular phononic crystals are also discussed. Numerical results demonstrated that all the optimized structures we obtained for cellular PnCs are similar to these obtained for composite case [20], except that additional slim connections are added in the cellular case, since they are essential to support the propagation of shear wave modes and to satisfy the prescribed bulk or shear modulus constraints. It is also found that the optimal relative band gap sizes dramatically decrease with the increase of stiffness constraint ratio while the slim connections in the corresponding topologies become thicker, which indicates that these connections can be slightly changed based on the manufacture precision on the cost of band gap size. Several optimized designs we obtained have broken the maximum band gap size record in the literature. The main difference between the final results with the bulk modulus constraint and that with the shear modulus constraint relies on the position of the slim connections.

## Abbreviations

The following abbreviations are used in this manuscript:

PnCs	Phononic crystals
FEM	finite element method
PWE	plane wave expansion method
TMM	transfer matrix method
MST	the multiple scattering theory
FDTD	finite-difference time-domain
RM	Rayleigh method
MMA	the method of moving asymptotes
GA	genetic algorithm
FPWE	fast plane wave expansion method
NSGA-II	non-dominated sorting-based genetic algorithm II
ESO	Evolutionary Structural Optimization
BESO	Bi-directional Evolutionary Structural Optimization
